# Optimizing use of an electronic medical record system for quality improvement initiatives in hemodialysis: Review of a single center experience

**DOI:** 10.1111/hdi.13178

**Published:** 2024-10-21

**Authors:** Noémie Laurier, Jorane‐Tiana Robert, Alexander Tom, Jerrica McKinnon, Nancy Filteau, Laura Horowitz, Murray Vasilevsky, Catherine Weber, Tiina Podymow, Andrey V. Cybulsky, Rita S. Suri, Emilie Trinh

**Affiliations:** ^1^ Faculty of Medicine and Health Sciences McGill University Montreal Quebec Canada; ^2^ Research Institute of the McGill University Health Center Montreal Quebec Canada; ^3^ Division of Nephrology McGill University Health Center Montreal Quebec Canada

**Keywords:** electronic medical record, hemodialysis, patient safety, quality improvement, review

## Abstract

**Introduction:**

The complexity of managing patients with end‐stage kidney disease on hemodialysis underscores the importance of implementing quality improvement (QI) initiatives to enhance patient safety and prioritize patient‐centered care. To address this, we established a QI committee at our tertiary academic center focusing on evidence‐based practices, patient‐centered approaches, and cost efficiency. To facilitate the seamless implementation of QI initiatives, we leveraged the capabilities of our electronic medical record (EMR) system.

**Methods:**

This review details effective strategies for optimizing use of an EMR system to successfully implement QI efforts. Drawing from our experience, we provide detailed descriptions and practical insights that can be applied to other EMRs.

**Findings:**

The creation of a secure and accessible dashboard, offering real‐time data on quality metrics, stands out as the most notable feature. This dashboard operates through an algorithm that merges data from both our dialysis and hospital EMR systems. Its primary objectives are to streamline the identification of high‐priority patients, enhance team communication, and facilitate tracking of quality indicators. Additionally, we integrated clinical pathways, checklists, and standardized protocols into the renal EMR to ensure smooth implementation of QI interventions. Notable examples of these interventions include an incremental hemodialysis protocol, a new hemodialysis start checklist, vaccination care plans, and personalized kidney transplant workups. Programmed electronic automatic reminders have proven invaluable in ensuring timely follow‐ups of assigned tasks. The EMR has also contributed to medication optimization and deprescribing by generating patient lists based on specific medication classes. Finally, the EMR's capability to swiftly generate lists of patients with specific features has significantly facilitated targeted QI interventions.

**Conclusions:**

Leveraging the capabilities of an EMR system can be crucial for enhancing care of hemodialysis patients and implementing effective QI initiatives.

## INTRODUCTION

Patients with end‐stage kidney disease on hemodialysis experience significant morbidity, mortality, and reduced quality of life.^1^, ^2^ Managing end‐stage kidney disease patients is complex, and quality improvement (QI) initiatives can play a crucial role in ensuring patient safety and delivering optimal patient‐centered care.^3^ Many successful QI initiatives in nephrology have been described in patients receiving conventional hemodialysis.^4^, ^5^, ^6^ For instance, a QI initiative in Ontario, Canada, demonstrated that reducing the frequency of blood work for stable chronic hemodialysis patients from once every 4 weeks to once every 6 weeks was time‐efficient, cost‐effective, and did not adversely affect clinical outcomes.^4^ Another QI intervention showed an increase in living kidney donor rates with the adoption of a 1‐day donor evaluation process.^5^ While these initiatives highlight the importance of QI interventions, the literature on QI initiatives in nephrology and hemodialysis is limited, highlighting the need to develop new QI interventions.^6^, ^7^, ^8^

Our health center is a tertiary care academic center with over 400 patients receiving chronic hemodialysis. To improve quality of care, we established a QI committee with multidisciplinary team members. The committee aims to standardize evidence‐based, high‐quality hemodialysis practices across sites, prioritize patient‐centered approaches, and optimize cost efficiency.^9^ Many of our QI initiatives have successfully leveraged our renal electronic medical record (EMR) system, *Renal Insight* (*Constellation Kidney Group*). This system is utilized by all renal nurses, physicians, and allied health team members in our center and is integrated with the hospital's main EMR, enabling automatic updates from various data sources. It includes essential clinical data such as dialysis session details, laboratory results, medical history, medications, and care plans. Additionally, it facilitates team communication, data tracking, and generates clinical reports. This integration has significantly contributed to the success of our QI initiatives. By streamlining communication and data management, the optimal use of our EMR supports our commitment to delivering high‐quality patient‐centered care efficiently and effectively.

As highlighted by Navaneethan et al., “future studies should explore the optimal methods of using electronic health records to improve chronic kidney disease care and research at the individual patient level, health system and population levels.”^10^ This review presents our experience with utilizing the novel functionalities of an EMR system to implement hemodialysis QI initiatives successfully and provide optimized patient‐centered care. This article aims to provide a comprehensive overview of these efforts, focusing on the following functionalities: creation of a QI dashboard, integration of clinical pathways, implementation of standardized protocols, medication optimization, facilitation of deprescription, rapid identification of high‐risk patients, and combined use of multiple functionalities for QI methodology (Table 1).

**TABLE 1:** Summary of the quality improvement initiatives facilitated by our electronic medical record (EMR).

EMR functionality	Clinical tools	Limitations
Quality improvement dashboard	Accelerates identification of patients requiring special attention	Absence of predictive analytics
	2 Improves communication within the health care team	
Clinical pathways and checklists	Facilitates delivery of effective patient care	Informatics specialist support necessary for creation and integration of new clinical pathways and/or checklists
	2 Standardizes and optimizes patient care	2 User training necessary for proper usage of this functionality
	3 Avoids oversights	
	4 Reduces administrative delays and paperwork	
Standardized protocols	Streamlines clinical care	Generated alerts must be interpreted by the treating staff to avoid medical errors
	2 Integrates evidence‐based medicine into clinical practice	
	3 Identifies patients falling outside the parameters of a protocol	
Medication optimization and deprescription	Optimizes patients' pharmaceutical lists	Security issues must be addressed before connecting the EMR system with pharmacy data
	2 Creates opportunities to deprescribe unneeded medications	
High‐risk patients identification	Generates categories and lists of high‐risk patients	Informatics specialist support necessary for creation of high‐risk identification filters
	2 Facilitates implementation of targeted interventions	
	3 Avoids oversights	
	4 Expedites delivery of care during unexpected events	
Combined use of EMR functionalities	Facilitates conduction of PDSA cycles	Requires understanding of QI methodology for proper interpretation
	2 Facilitates conduction of Root Cause Analyses	

Abbreviations: PDSA, Plan‐Do‐Study‐Act cycle; QI, quality improvement.

## METHODS

We reviewed our local experience using an EMR to enhance patient‐centered care and implement successful hemodialysis QI initiatives. Qualitative feedback was obtained directly from key stakeholders, including patients, health care providers, QI committee members, and a clinical informatics specialist. This feedback allowed us to thoroughly describe the strengths and limitations of each EMR functionality. We carefully selected EMR algorithms that are easily applicable and particularly useful in nephrology and hemodialysis. Furthermore, quantitative data were collected longitudinally directly through the EMR to evaluate the effectiveness of our implementations, including the number of patients involved and the completion rates of specific initiatives. Drawing from our experience, we provide detailed descriptions and practical insights that can be applied to other EMRs.

## KEY FUNCTIONALITIES

### Creation of a dashboard with real‐time reports of QI metrics

**FIGURE 1:**
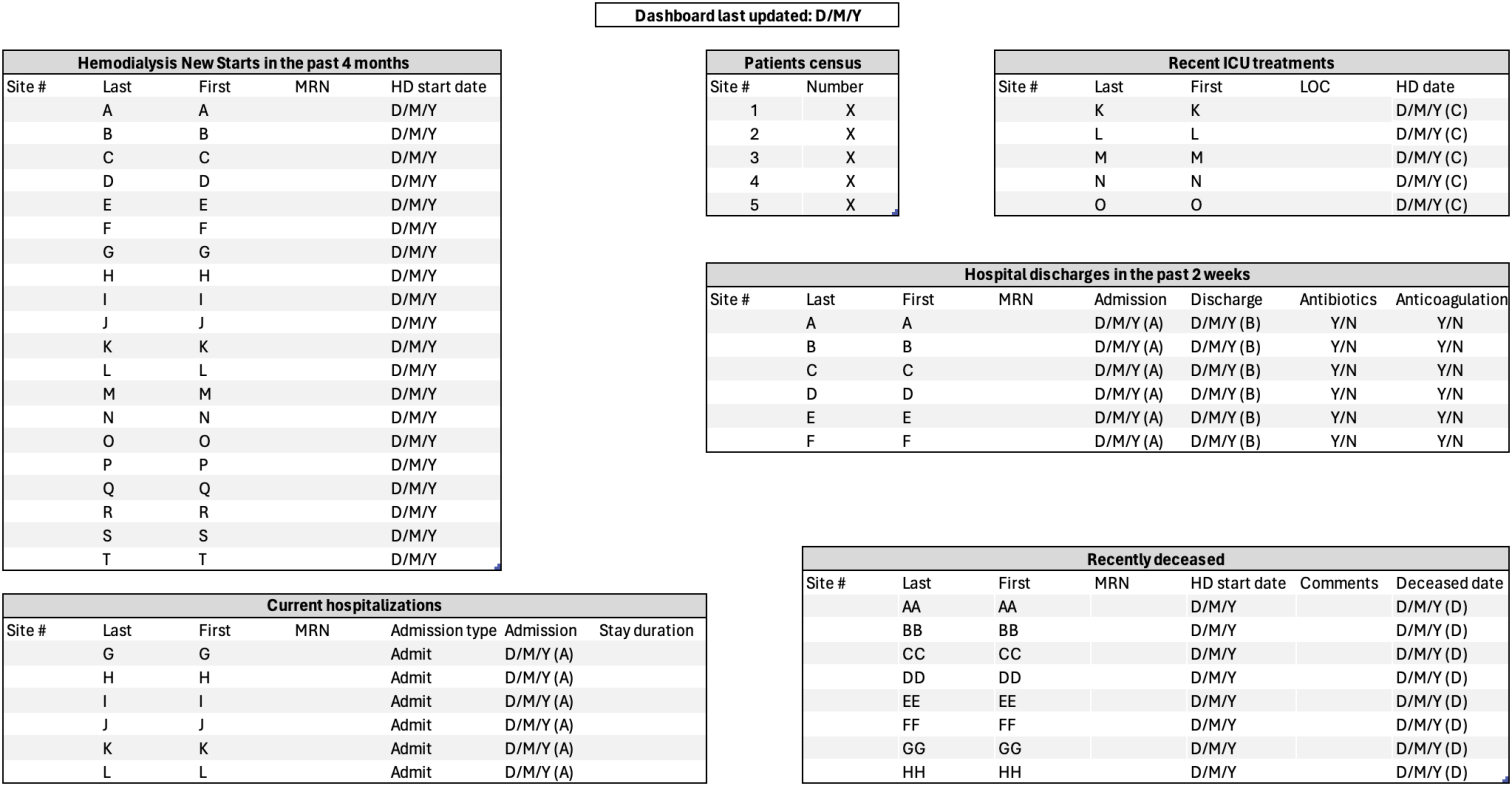
Example of our quality improvement dashboard. HD, hemodialysis; ICU, intensive care unit; LOC, level of care; MRN, medical record number.

Through a one‐time programming using an algorithm combining real‐time data from our dialysis EMR and the hospital EMR, we created a self‐updating dashboard containing various QI metrics for patients receiving hemodialysis (Figure 1). This approach reflects a growing global strategy aimed at ensuring proper monitoring of quality of care.^11^ Accessible through a security‐first strategy, this dashboard enables rapid tracking of crucial quality metrics, facilitating timely targeted interventions. It also facilitates team communication by providing easily accessible and secure information, minimizing reliance on redundant emails and/or verbal communications.

The development of this self‐updating dashboard was achieved through filters that identify patients on hemodialysis with specific clinical or laboratory profiles, generating clinical care cohort lists. Examples of filters include patients newly started on dialysis, home dialysis prevalence, recent hospital discharges, intensive care unit admissions, vascular access proportions, transplant listing, and patients prescribed at‐risk medications such as anticoagulants and antibiotics. Access to the dashboard helps improve time efficiency in clinical practice by allowing rapid identification of patients requiring special attention. It reduces the need for individual patient chart review to identify a subset of patients with health indicators of concern. As a result, clinical care and patient safety are improved, allowing for timely and targeted interventions. It should be noted, however, that the simplicity of the dashboard's programming restricts its capabilities, as it cannot perform predictive analyses.

### Integration of clinical pathways and checklists

Another valuable QI tool facilitated by an EMR system is the integration of clinical pathways. Clinical pathways provide recommended steps to the treatment team based on the desired treatment or care plan. These enhance the effectiveness of patient care, while reducing variability in clinical practice aligned with the most recent literature.^12^ Furthermore, through frequent reassessments and Plan‐Do‐Study‐Act (PDSA) cycles, where knowledge is gained at each step,^13^ these pathways can be revised and modified to ensure optimal care and contribute to the success of QI interventions.

The integration of clinical pathways into our EMR has optimized care efficiency by embedding all necessary steps and tasks into the software interface. For example, patients who start on an incremental hemodialysis prescription require careful monitoring for the development of uremic symptoms or fluid overload in case of residual kidney function decline.^14^ To address this, an incremental hemodialysis clinical pathway with clear criteria was created and integrated into the EMR. When an incident patient is deemed eligible for incremental hemodialysis, all necessary prescriptions and tests are automatically ordered, and reminders are sent to the relevant team members at routine time intervals according to the clinical pathway. Contraindications to a continued incremental prescription are integrated, with a clinical checklist automatically prompted every 6 weeks for the bedside dialysis nurse to complete and the physician to review. Since November 1, 2022, 18 patients remain on an active incremental hemodialysis care plan.

**FIGURE 2:**
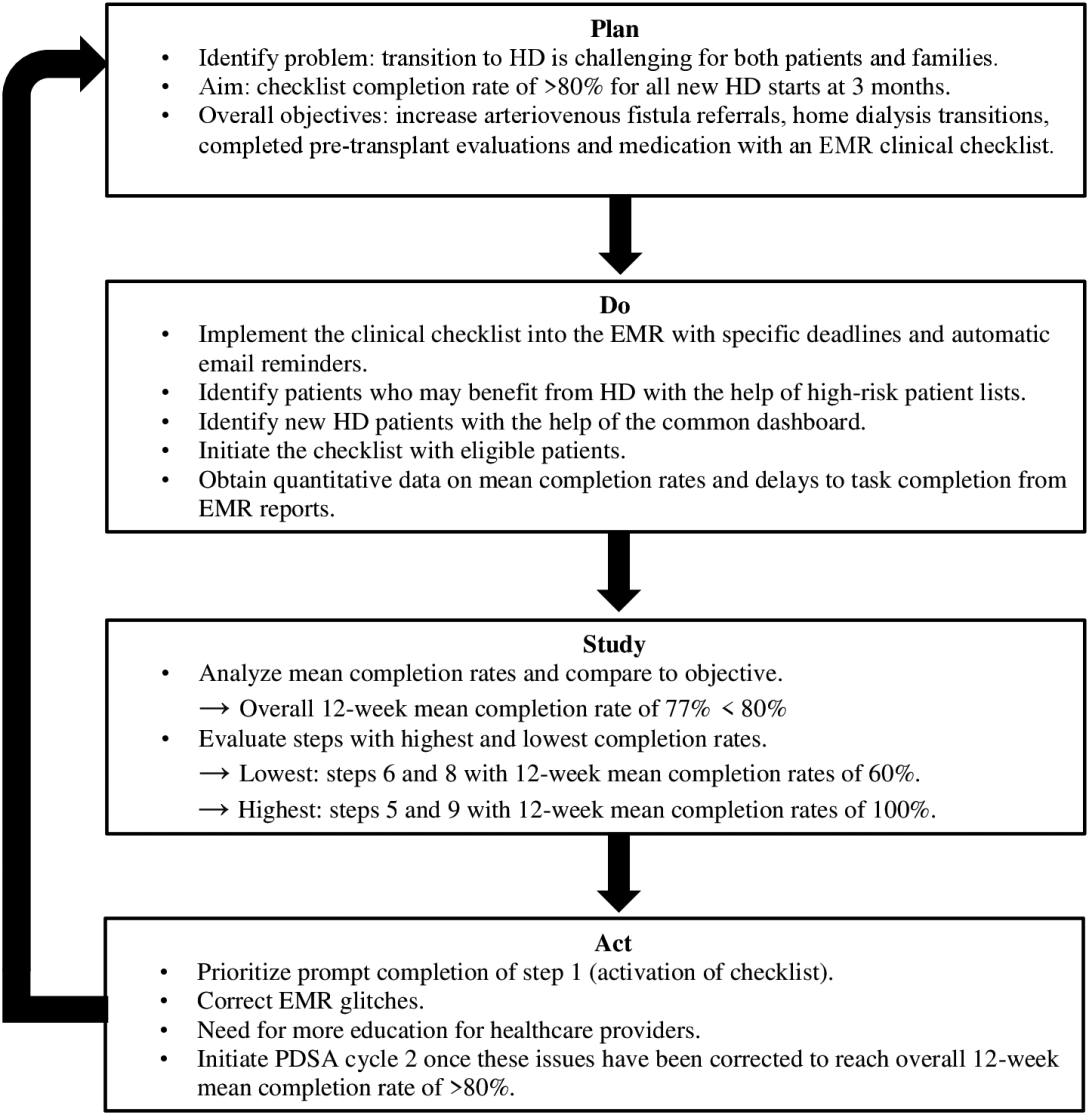
First Plan‐Do‐Study‐Act cycle of the “New Hemodialysis Start” clinical checklist, facilitated by use of multiple electronic medical record functionalities. EMR, electronic medical record; HD, hemodialysis; PDSA, Plan‐Do‐Study‐Act cycle.

Furthermore, the transition period to the start of hemodialysis is associated with an increased risk of adverse clinical outcomes and impaired quality of life.^15^ In September 2023, we implemented, through our EMR, a simple “New Hemodialysis Start” clinical checklist (Table 2) to ensure comprehensive care during this high‐risk transition period.^16^ Between September 2023 and April 2024, 27 new hemodialysis patients benefited from this QI initiative, and the overall 12‐week mean completion rate was 77% (Figure 2). Other clinical pathways that have been implemented include vaccination care plans and detailed patient‐centered transplant workups to facilitate and expedite care delivery processes. The vaccination care plans include complete immunization lists with descriptions of required doses and special indications for each vaccine. Similarly, a transplant evaluation checklist was integrated into the EMR with detailed indications for each potential test.

**TABLE 2:** New starts clinical checklist integrated into the renal electronic medical record (EMR) system.

Item	Team member assigned	Completion
Primary Physician Assignment	Assistant nurse manager	☐
2 Level of Intervention	Primary physician	☐
3 Detailed History	Primary physician	☐
4 Medication List	Primary physician	☐
5 Modality Education	Clinical nurse	☐
6 Vascular Access Review	Vascular access nurse	☐
7 Transplant Evaluation	Primary physician	☐
8 Allergies Review	Primary physician	☐
9 Social Worker Consultation	Social worker	☐
10 Dietician Consultation	Dietician	☐

*Note*: Inclusion criteria: (1) Patients with progressive chronic kidney disease starting on in‐center hemodialysis. (2) Patients with acute kidney injury remaining on hemodialysis after 90 days.

In summary, the integration of clinical pathways into an EMR can help ensure comprehensive, standardized patient care and help reduce administrative delay and paperwork. Importantly, the entire treating team can readily view completed and outstanding tasks, optimizing coordination to ensure timely provision of care. The only limitation is the need for support from an experienced clinical informatics specialist for their creation, integration, and modification.

### Facilitating implementation of standardized protocols

To facilitate the implementation of standardized protocols, a simple analytic model can be built into the EMR to streamline clinical care. This allows the EMR to analyze data in real‐time and provide prompts for optimal actions. The system can be programmed to notify staff about necessary changes in drug dosage, new test orders, and treatment modifications. In fact, a recent systematic review reported that 69% of studies evaluating clinical outcomes noticed improvement in quality of care after incorporating analytic models into their EMR systems.^17^

One example at our center is the initiation of a high‐dose proactive intravenous (IV) iron dosing strategy. In 2019, we implemented this proactive IV iron strategy based on results from the Proactive IV Iron Therapy in Hemodialysis Patients  study^18^ and feedback from key stakeholders. The protocol was integrated into our EMR with a clear algorithm informing the administration of IV iron — 400 mg of IV iron sucrose every 6 weeks if hemoglobin is <126 g/L, transferrin saturation <40%, and ferritin ≤700 μg/L. The model generates specific alerts and actions according to its analysis. Therefore, if the patient meets criteria for high‐dose IV iron administration, the model will alert the primary physician. Conversely, if the patient's results fall outside the set parameters, the model will indicate the need to hold the IV iron until the next laboratory cycle (every 6 weeks).

While EMR notifications must be interpreted with caution and final decisions made by the treating health care providers, analytic models can help ensure a seamless and standardized process. They improve quick identification of patients who fall outside the parameters of a designated protocol, thus avoiding potential adverse clinical outcomes.

### Medication optimization and deprescribing opportunities

Optimal use of our EMR has greatly facilitated medication optimization and deprescription opportunities. Safe deprescribing is an emerging area of interest in QI, particularly due to the common issue of polypharmacy among elderly patients and those with chronic illnesses.^19^ Polypharmacy is associated with increased morbidity, frailty, and diminished physical and mental well‐being.^20^ A recent cross‐sectional study revealed that patients on dialysis typically receive an average of 14 ± 4.6 medications, which significantly exceeds the threshold for polypharmacy classification of ≥4 to 5 medications.^21^ Importantly, a higher amount of medication prescriptions has been associated with a reduced quality of life in patients on dialysis.^22^ This is primarily due to increased medication administration demands interfering with daily activities, drug interactions, and reduced comprehension of the medications' needs by the patient, potentially leading to non‐adherence.^22^ This underscores the value of deprescribing QI initiatives.^23^, ^24^

At our center, all medications for patients on hemodialysis are updated directly into the renal EMR routinely every 6 months and after every hospitalization. This regular review schedule enhances the quality of care associated with safe prescribing. Indeed, the electronic recording of medications facilitates identification of all patients taking specific medications or classes of medications. As such, this feature allows for the implementation of interventions to review pharmacologic indications, optimize dosing, and/or create opportunities to deprescribe. As examples, we have used our EMR to generate reports on patients who remain on immunosuppression, anticoagulants, and proton‐pump inhibitors to review the clinical indications for use or assist with regular monitoring of serum drug levels. We have also used our EMR to identify patients on loop diuretics to target optimal dosing or deprescribe with loss of residual kidney function.

Finally, more optimal pharmacologic QI initiatives will be possible in the future as we work on directly connecting our EMR system with inpatient and outpatient pharmacy data to allow real‐time up‐to‐date medication information. However, several security issues will need to be addressed before creating this interface.

### Rapid identification of high‐risk patients

#### In a typical clinical setting

Integration of relevant clinical and laboratory parameters into our EMR allows rapid identification of hemodialysis patients at high risk of adverse clinical outcomes through export of data into clinical reports. Examples of reports generated include: patients at high risk of fluid overload (i.e., with high interdialytic weight gains, high ultrafiltration rates, and/or frequent intradialytic hypotension), patients with severe hyperkalemia, and patients with frequent non‐adherence. The EMR can also provide rapid reports on specific categories of hemodialysis patients, such as recent dialysis starts, recent hospitalizations, transplant candidacy categories, patients on specific medications, or patients with specific dialysis parameters. These lists are useful reminders, signaling the potential necessity for extra attention. Finally, targeted alerts about high‐risk infections and the need for isolation precautions have been incorporated in the EMR. Alerts include immunosuppressed hemodialysis patients newly diagnosed with Coronavirus infection 2019 (Covid‐19) who might benefit from timely adjuvant antiviral therapies and patients with multi‐drug resistant organism infections. Between 2020 and December 31, 2022, 39 alerts were sent by the EMR for newly diagnosed Covid‐19 hemodialysis patients on immunosuppressive medications.

Rapid identification of high‐risk patients is essential in QI because it enables the effective implementation and monitoring of targeted interventions. By carefully selecting patients who would benefit most, QI efforts become more impactful.

#### In a natural disaster setting

Another pertinent example of how our renal EMR has been crucial in facilitating efficient optimal targeted care was in the setting of recent Quebec, Canada, wildfires in the summer of 2023. Wildfires led to the displacement of many rural patients receiving dialysis from their communities and led to their temporary transfer to our urban dialysis units. Given the increasing frequency of natural disasters and other unexpected events due to climate change, it is critical to proactively anticipate their impact on the health care system. Such predictive needs apply to many treatments, including hemodialysis. In the event of a catastrophe, health providers must have the necessary resources to rapidly identify high‐risk patients who cannot forgo dialysis sessions and for whom emergency solutions should be established.

Recently, forest fires, power failures, and a major water leak leading to the temporary closure of a dialysis unit highlighted the need to rapidly identify high‐risk patients in our urban and satellite dialysis centers. Although health care workers possess the capability to independently identify these patients, our EMR system increases the speed of identification and of subsequent action. Our clinical informatics specialist created new filters for risk stratification, which identify patients at high risk of fluid overload, who have high interdialytic weight gain, and with severe hyperkalemia. The high‐risk patients require more rapid transfers to other dialysis centers under such circumstances. In fact, during the Canadian wildfires, 63 hemodialysis patients living in Quebec James Bay Cree communities were identified by the filters and rapidly transferred to dialysis centers in unaffected Quebec regions. Additional filters helped generate lists of patients with acceptable health indicators who could temporarily decrease their number of weekly dialysis sessions, thereby allowing for accommodation of high‐risk patients if dialysis space was limited.

### Combined real‐time data collection and analysis

When used comprehensively, EMR functionalities can significantly enhance conduction of QI methodology. They can provide real‐time data on team members' contribution, delays in task completion, patients' health status, and other aspects of patient care. Additionally, these functionalities aid in completing Root Cause Analyses to identify barriers to the success of specific interventions.^25^ For each QI initiative embedded into the EMR, our local clinical informatics specialist generates real‐time reports with pertinent quantitative data points. These longitudinal data have been invaluable in our PDSA cycles that are conducted to analyze the achievability, limitations, and strengths of QI initiatives.

Furthermore, longitudinal qualitative data is collected from key stakeholders. This has been done via informal feedback during quarterly QI division‐wide rounds and through semi‐structured interviews with thematic analysis. For example, following the implementation of our incremental hemodialysis protocol, we asked specific questions to select patients and health care providers (Table S1). Consequently, the combined qualitative and quantitative data provides opportunities to adapt interventions within PDSA cycles and obtain input and perspective from key stakeholders (Figure 1).

### Applicability to other dialysis centers and EMRs

The described functionalities can be incorporated and adapted into other EMR systems with the assistance of a clinical informatics specialist. Real‐time dashboards, for instance, can be easily implemented through the creation of filters relevant to specific nephrology sites. Recently, Cincinnati Children's Hospital Medical Center created a continuous renal replacement therapy quality dashboard, and it was successful in monitoring the process and quality of this therapy.^26^ Additionally, most hemodialysis centers update patients' medication lists directly within their EMR, providing opportunities to rapidly identify patients on select medications. The checklists described in this review can also be adapted to any hemodialysis center and added to their EMR. For example, a study in Boston added a “Chronic Kidney Disease  checklist” to their EMR, facilitating management of chronic kidney disease patients in primary care.^27^ Another study conducted in the United States used an EMR analytic model to predict chronic kidney disease progression and taper renal care accordingly.^28^ This model included data such as age, gender, comorbidities, and estimated glomerular filtration rate, aiding health providers in decision‐making. This example highlights the potential for the implementation of analytic models in many hemodialysis centers. Finally, in an emergency setting, the use of an EMR has proven to be timely and efficient at our tertiary care center and this strategy could be widely adopted to improve preparedness for environmental disasters.

## LIMITATIONS

The EMR system presented in this article is used in a single academic center and its affiliates, and thus the described examples may have limited generalizability. Additionally, the addition of novel functionalities within the EMR system is dependent on an experienced clinical informatics specialist. This may be a laborious and time‐demanding task and can lead to delays in QI interventions that rely on the use of the EMR system to be operationalized. Finally, as with any informatics system, this renal EMR is imperfect, and errors are possible when generating automatic task reminders, action alerts, and patient lists. As such, health care professionals must keep this in mind while reviewing the EMR system's output.

## KEY LEARNINGS

The main driver of success in our QI initiatives was the collaborative effort of a multidisciplinary team, anchored by a dedicated informatics specialist. Through this teamwork, we identified QI needs, which the informatics specialist addressed by proactively optimizing EMR functionalities. This collaboration enabled the swift incorporation of new algorithms and ensured the sustainable implementation of multiple initiatives, demonstrating the immense potential of a collaborative approach to EMR optimization. The opportunities for EMR optimization are vast, making this strategy a valuable asset for implementing multiple sustainable actions.

## CONCLUSIONS

This article has detailed how a renal EMR system, enhanced by inclusion of novel functionalities, facilitated the implementation of numerous QI initiatives to optimize hemodialysis at a single academic center and its affiliates. The use of an EMR can facilitate the process of routine data analyses through regular PDSA cycles to enable more refined solutions. Use of an EMR can also help identify patients who would most benefit from targeted QI interventions and helps prioritize meaningful actions. Finally, notifications embedded within the EMR can prompt timely completion of tasks with minimal oversight, thus increasing the chances of sustainable QI interventions. In summary, we have described how special features of an optimized renal EMR can be used to promote QI driven care with a collaborative multidisciplinary approach aimed at improving patient management and experience. The described EMR functionalities can serve as models for QI teams who wish to accelerate completion of tasks and increase chances of sustainable interventions.

## CONFLICT OF INTEREST STATEMENT

Emilie Trinh has received speaker honoraria from Davita and Baxter and investigator‐initiated research funding from Otsuka. All other authors declare no conflict of interest.

## Supporting information


**Table S1.** Patients and health care providers perspectives on an incremental hemodialysis protocol.

## Data Availability

The data that support the findings of this study are available on request from the corresponding author. The data are not publicly available due to privacy or ethical restrictions.
