# Supplementary material for: Optimizing use of an electronic medical record system for quality improvement initiatives in hemodialysis: Review of a single center experience
Source: Hemodial Int. 2024 Oct 21;29(1):74–82. doi: 10.1111/hdi.13178 (PMC11729301; doi:10.1111/hdi.13178)
Supplement: Supplementary file 1 — Table S1. Patients and health care providers perspectives on an incremental hemodialysis protocol. [file HDI-29-74-s001.docx]

**Supplementary Material**

**TABLE 1.** Patients and Healthcare Providers Perspectives on An Incremental Hemodialysis Protocol.

| Questions |
| --- |
| 1. **Patients:** |
| 1. How did you first learn about twice a week dialysis? |
| 1. What were your initial thoughts about twice a week dialysis? |
| 1. What were your thoughts about starting hemodialysis twice per week, as opposed to three times per week? |
| 1. How would coming to dialysis three times per week, instead of two-times, have affected your life? |
| 1. In what ways do you think the transition process to starting dialysis would have been different if you had started three times per week instead of two? |
| 1. Would you change anything if you could restart the process? |
| 1. **Healthcare Providers:** |
| 1. How was incremental dialysis first presented to you as an option for patients? |
| 1. How were you trained to incorporate incremental dialysis into your practice? |
| 1. What are the challenges with an incremental dialysis protocol in your practice? |
| 1. What facilitates having an incremental dialysis protocol in your practice? |
| 1. Overall, how do you feel incremental dialysis impacts the patient experience? |
| 1. What has been the overall impact, for you, of an incremental dialysis protocol in your practice? |
| 1. What would you change in how incremental dialysis is presented or used with patients? |
